# Indents

**Published:** 1920-07

**Authors:** 


### INDENTS

☞ **Brief Articles of Technical or Scientific Value will receive notice and comment. Contributions invited.**

---

PULP CAPPING DRESSING. Equal parts of thymol and acetone, if applied directly to exposed pulps and covered with two coats of varnish, which are permitted to dry serially, will make a useful application and protection before applying plastic cement filling.—Herman Prinz.

---

TWENTY-FIVE MILLION pounds of food, medicines and clothing passed through the American Red Cross warehouses in the Balkans from January 1, 1918, to September 1, 1919. These supplies were valued at more than $10,000,000. Every ounce of this material came from the American people. Of the 40,000,000 souls in the Balkan states, more than 10,000,000 were beneficiaries of this bounty.

---

HYDROPAROTITIS FROM ARTIFICIAL TEETH. Jardet calls attention to a recurring swelling and pain in the parotid gland which is liable to develop when artificial teeth are worn. If the patient has happened to take out the plate when being examined, the physician may be misled.—*Journal A. M. A.*, June 12, 1920.

---

CASTING A RICHMOND USING STEELE'S FACING. Grind facing and backing to fit root end in front, sloping up to form a clearance of 1/32 of an inch in the back. The pin should be about 1/16 of an inch longer than canal, serrated on, and beveled from facing on projecting end. Oil end of root, mold inlay wax over same and melt to end of pin. Melt an appropriate amount of inlay wax to backing, oil gingival end of facing, and heat same enough to soften inlay wax on root when both are forced to place. The wax on root may be softened with spatula immediately before forcing facing and backing to adjustment. Unite two portions of wax with hot spatula, chill, remove, and carve wax; remove facing, invest, and cast. —Josep Homer, Boston, Mass.

---

A New Price for Drugs. Before the American Red Cross went into Vienna there were three prices for prescriptions and common drugs. Now there are four. There were different prices for the wealthy, the middle class and the poor. Now those in dire need receive drugs from the Red Cross, and the price to these is merely the asking. Supplies are also placed with druggists who pay for them only what they are able. This system of distribution is working admirably and the supplies are reaching the persons who need them most.

---

Why Some Gold Fillings Become Loose. When a gold filling is subjected to the impact of mastication for a time, it will undoubtedly change its shape, so that the fillings in a good many teeth will become loose, not because they are improperly shaped, not because the tooth has been improperly filled, but because of the impact brought to bear upon the gold. In time it is forced from position by means of a change in its shape.—W. H. T., in *Oral Health.*

---

Licensing Anesthetists. H. C. Macatee, recording secretary of the Medical Society of the District of Columbia, advises that at the May meeting of that organization, the following resolution was adopted:

*Resolved,* That the Medical Society of the District of Columbia go on record as in favor of the limitation of the practice of anesthesia to regularly licensed physicians and surgeons and dentists and graduate nurses in cases of emergency, or medical students for the purposes of instruction.

PROFESSOR OF ANESTHESIA. Dr. S. Griffith Davis, of the Research Committee of the National Anesthesia Research Society, advises that the University of Maryland, which several years ago created a separate department of anesthesia, and put him in charge with the title of associate professor, has now realized the importance of the work and given him a full professorship. So far as records are available, this is the first professorship of anesthesia to be created in the United States. Dr. Davis ventures to hope that other schools will soon follow the example set by the University of Maryland.

---

ANESTHETISTS. The executive secretary is privileged to announce the addition of two of the most eminent scientists in America to the membership of the Research Committee of the National Anesthesia Research Society. These are Dr. Yandel Henderson, of Yale University, and Dr. Charles H. Baskerville, of the University of New York City. The Society has also received the membership of several distinguished doctors, surgeons and anesthetists of the Dominion of Canada.

---

THE RED CROSS ROLL CALL. The Fourth Red Cross Roll Call will be held from Armistice Day, November 11th, to Thanksgiving Day, November 25th, next. Hereafter every anniversary of the end of hostilities in the World War will be the occasion for the American public to renew its Red Cross allegiance through dollar memberships.

This was made known recently by Dr. Livingston Farrand, chairman of the Central Committee, when he announced that as a result of the last roll call the Red Cross now has more than ten million members. This is more than twenty times the pre-war membership of the Society, and does not take into account the fourteen million school children who are members of the Junior Red Cross.

The membership dollars will be used to further the gigantic peace-time activities of the American Red Cross, which are:

*To continue work for America's veterans of the World War, particularly the disabled.*—At the Red Cross Institute for the Blind, near Baltimore, more than half of the American soldiers blinded in the World War have already been trained for living and earning without their sight.

*To serve our peace-time army and navy.*—The Government has requested that the Red Cross continue this responsibility, particularly that of acting "as a connecting link between the enlisted men and their families."

*To develop stouter national resistance to disease through health centers.*—The Red Cross Chapter in Seattle, Wash., alone is establishing twenty-five Red Cross health centers in the towns of King and Kitsap counties.

*To increase the country's nursing resources and to co-operate with official health agencies.*—When influenza visited New York City, the Red Cross supplied 12,600 blankets, towels, nightgowns, layettes, and other sick-room articles within a few hours. In Chicago, 14,000 women trained by the Red Cross during the war were called to sick-room service.

*To continue preparedness for disaster relief.*—Mobile relief units, consisting of food and medical supplies, are stored in Red Cross warehouses all over the country. In time of disaster they can be rushed to the stricken community.

*To continue home service and community work.*—Red Cross home service workers are in forty-five U. S. public health service hospitals, with a possible population of 10,000 patients.

*To complete relief work among the war-exhausted and disease-ridden people of Europe.*—Ten millions of the 40,000,000 souls in the Balkan states alone were beneficiaries of Red Cross bounty in seventeen months of relief work there. The food and clothing and medical relief supplied are given as "gifts of the American people."—*American Red Cross*, July, 1920.

---

TRAINING DENTAL HYGIENISTS. Between two and three hundred children are treated daily, being taught, besides, that a clean tooth never decays, that proper nourishment, thorough mastication and a clean mouth insure physical and mental development. And it is for the prophylactic work, of course, that we have the "Forsyth Dental Hygienists Training School," which offers unusual opportunities to the student of dental hygiene.

The facilities of the various clinics and laboratories of the infirmary are available and all of the girls receive thorough and comprehensive training which will fit them for three distinct types of positions:

*First.* They may be teachers in public schools and institutions.

*Secondly.* They may operate as dental hygienists in the prophylactic treatment of the teeth.

*Thirdly.* They may act as dental nurses in private offices and institutions.

Throughout the course theory and practice go hand in hand.—Anna V. Hughes, in *Cosmos.*

---

PHARMACOLOGICAL ACTION OF SILVER NITRATE. We are especially interested in the pharmacological action of silver nitrate. I deduce from his writings that Dr. Howe emphasizes the fact that silver is the vital factor. Metals themselves do not exercise any pharmacological function. If by accident a child takes a copper penny in the mouth and swallows it, in the great majority of cases it passes through the intestines without causing any harm. In bygone days it was rather common practice in cases where there was some obstruction in the intestines, to give the patient about two pounds of mercury. It passed through the intestinal canal without causing poisonous effects.

The pure silver metal does not exercise any therapeutic function. When we employ a metallic salt, it is the acid radical which produces the effect; the metal is merely the carrying medium of this basic factor.

If you have a four-wall cavity, and you place in the cavity silver nitrate, and immediately cover it with temporary stopping, and let your patient go—when the patient returns in two or three days the cavity is not black, it is lemon yellow, because there was no chance for the light to act on the liberated nitric acid radical. Expose the silver nitrate to light, and it will turn black, provided chlorine is present. As we know, chlorine in the form of sodium chloride is present in all the tissues of the body. The metal (silver) in silver nitrate is merely the carrying factor of its therapeutic ion—nitric acid. Let me illustrate this further. We have a compound known as collargol. It contains about 86 per cent. of silver in a very fine state of division, while silver nitrate contains about 68 per cent. of silver. But silver nitrate is at least fifteen times stronger in its antiseptic power than the former, because the very finely powdered silver is only in a very limited degree changed to a silver albumin chloride which, in turn, is ionized.

Dr. Howe has shown beautiful specimens of deep penetration of silver nitrate into the tubuli. The albumin becomes precipitated and solidly plugs up these tubuli, and any further action of micro-organisms is cut short. The only drawback is the discoloration of the dentine.

I have my doubts as to whether you can apply this principle in the treatment of the teeth of adult patients in your practice. In using dichloramin-T for the same purpose, as I have outlined in a recent number of *Dental Cosmos,* you will get the same results minus the discoloration. In filling root canals the important point is to prevent reinfection. The blocking of the dentinal tubuli is the only absolutely safeguard to prevent this secondary infection.—Herman Prinz, in *Items of Interest.*

A Salivary Deposit Solvent or Remover. Do not get the idea that the application of the following will remove such deposits without instrumentation. It will, however, assist in several ways. With its use the deposit will not stick as tenaciously, and the underlying soft tissues will have a better tone. The danger of causing post-operative inflammation is also reduced by the application of equal parts of campho-phenique and tincture of iodine once or twice before the instrumentation is to take place. —*F. W. F.*

---

Gingival Irritations. The more carefully we study the gingiva the more we come to realize that the majority of cases of gingivitis are due to continuous or often repeated irritation. We should consider the gingiva as guarding the peridental membrane against infection. The gingiva are equipped with every facility for prompt repair; they will withstand an enormous amount of punishment and offer the most vigorous resistance. We have failed to do our part in helping the gingiva to protect the peridental membrane. In the care of a mouth, I would rather overlook a cavity than an inflammation of a gingiva. Most areas of gingivitis are easily cured by simple operations; the difficulty is that we have not recognized the fact that preventive treatment of peridental disease must be applied to inflammations of the gingiva, which necessarily precede. It must come to be a part of the regular routine of our mouth examinations to make a record of every area of gingivitis and to do whatever may be necessary to cure these inflammations before the peridental membrane is involved. When we come to do this, we will have really begun preventive treatment of peridental disease.—Dr. A. D. Black, in *Journal N. D. S.*

### WHY DROWN?

The best way to drown is to throw up both hands and holler "Help!"

---

The small boy makes a good life saver because he has not learned fear, and three of him are usually present before the first adult arrives on the scene.

---

If a duck had to wear shoes and stockings he would never have learned to swim.

---

Lots of good barrels have been ruined rolling drowned people over them. Learn the proper method of resuscitation.

---

Fellows, never take a girl canoeing unless you can swim for two, or know a girl that could save you.

---

A canoe is different from a canal boat. It is safe to stand up in a canal boat.

---

If clutched by a drowning person, encourage him to climb up and get from under as rapidly as possible.

---

A small boy is a good life saver, for, like a tugboat, he has the engine power without the cargo space.

—*Red Cross.*

